# Epidemiological analysis of anaplasmosis in cattle from Khyber Pakhtunkhwa, Pakistan

**DOI:** 10.14202/vetworld.2023.2287-2292

**Published:** 2023-11-19

**Authors:** Farhad Badshah, Kalim Ullah, Mustafa Kamal, Naseem Rafiq, Tahir Usman, Patricio R. De los Ríos-Escalante, Mourad Ben Said

**Affiliations:** 1 Department of Zoology, Abdul Wali Khan University, Mardan, Pakistan; 2 Department of Zoology, Kohat University of Science and Technology, Kohat-26000, Pakistan; 3 College of Veterinary Sciences and Animal Husbandry, Abdul Wali Khan University, 23200 Mardan, Pakistan; 4 Universidad Católica de Temuco Facultad de Recursos Naturales Departamento de Ciencias Biológicas y Químicas Casilla 15-D Temuco, Chile; 5 Nucleo de Estudios Ambientales, UCTemuco, Chile; 6 Department of Basic Sciences, Higher Institute of Biotechnology of Sidi Thabet, University of Manouba, Manouba 2010, Tunisia; 7 Laboratory of Microbiology, National School of Veterinary Medicine of Sidi Thabet, University of Manouba, Manouba 2010, Tunisia

**Keywords:** anaplasmosis, biotic risk factors, cattle, Khyber Pakhtunkhwa, Pakistan, spatiotemporal variation

## Abstract

**Background and Aim:**

Anaplasmosis, a tick-borne disease affecting livestock caused by the bacteria *Anaplasma*, poses a global concern. This study aimed to estimate the prevalence, spatiotemporal variation, and associated risk factors of anaplasmosis in cattle from the Bannu and Lakki Marwat districts of Khyber Pakhtunkhwa, Pakistan.

**Materials and Methods:**

This study used 197 cattle exhibiting clinical symptoms of anaplasmosis in natural settings. Microscopic examination was used to estimate the prevalence. Potential risk factors, such as sampling regions and months, gender, breed, and age were studied.

**Results:**

The study revealed an overall anaplasmosis prevalence of 19.79%. Bannu district exhibited a higher occurrence at 22.10%, compared to Lakki Marwat district at 17.64%. Young cattle (<2 years) demonstrated a notably higher incidence of anaplasmosis (26.78%) compared to adults (>5 years), which had a prevalence of 12.35% (p < 0.05). Female cattle (22.36%) were more susceptible than male cattle (11.11%). Prevalence peaked in June (45.71%) and was lowest in February (3.57%). Crossbred cattle had a higher prevalence (23.52%) than purebred cattle (11.47%).

**Conclusion:**

Anaplasmosis can be effectively controlled using a comprehensive approach encompassing selective breeding for resilience, targeted care of young calves and females, effective tick control during warmer months, consistent use of insecticides, and proactive risk factor management. Raising awareness among farmers through diverse channels, including media, is pivotal to bolster tick-borne disease management strategies.

##  Introduction

Anaplasmosis, classified as gall sickness, is a tick-borne disease caused by Gram-negative, obligate intracellular, spherical bacteria of the *Anaplasma* genus. These micro-organisms reproduce within vacuoles created from cell membranes in vertebrate or invertebrate hosts [1]. Anaplasmosis can affect humans and various domestic and wild animal species, including dogs, horses, goats, sheep, cats, ruminants, birds, and other fauna, resulting in the spread of this disease [2, 3]. The primary causative agent of anaplasmosis in cattle is *Anaplasma* *marginale*, which causes acute anaplasmosis, resulting in severe morbidity with global impact [4]. Bovine anaplasmosis, prevalent in economically disadvantaged regions, inflicts significant financial burdens due to reduced production, weight loss, expensive treatments, and even fatalities and abortions [5]. The economic ramifications of anaplasmosis are profound, often resulting in local and global trade restrictions [6]. Reports indicate widespread occurrence of anaplasmosis in cattle and buffaloes with the following prevalence rates: Bangladesh (25.82%) [7], India (11%) [8], Sudan (13.87%) [9], Iran (37.3%) [10], China (3.2%) [11], and Morocco (21.9%) [12]. In Pakistan, anaplasmosis has been reported in Lakki Marwat (19.66%) [13], Lodhran (9%), Dera Ghazi Khan (17%) [14], and Faisalabad (10.84%) [15].

Animal husbandry significantly contributes to Pakistan’s economy, serving as the primary source of income and sustenance for 8 million rural families [16]. As an agrarian nation, the animal husbandry sector has substantially progressed in Pakistan, contributing to 58.92% of the agricultural sector. The cattle industry has a considerable economic impact but faces considerable challenges due to infectious and non-infectious ailments. Highly transmissible tick-borne infections impose significant financial burdens on farmers. Among these, anaplasmosis is a prevalent tick-borne bacterial disease affecting domestic animals, resulting in substantial economic losses [16].

This study aimed to estimate the prevalence of anaplasmosis in the Bannu and Lakki Marwat districts of Khyber Pakhtunkhwa, Pakistan, considering its spatiotemporal variations. Further, the potential biotic risk factors associated with anaplasmosis, such as cattle age, gender, and breed, were identified.

## Materials and Methods

### Ethical approval

This study is a part of the research work conducted by the first author during his bachelor’s degree in Zoology at Kohat University of Science and Technology, Kohat-26000, Pakistan. It is pertinent to mention that ethical approval from the Ethical Committee of Kohat University of Science and Technology was not required for conducting undergraduate research work at that time. All procedures were performed by qualified professionals with a commitment to animal welfare. Informed consent was obtained from cattle owners before the collection of blood samples from their animals.

### Study period and location

**Figure-1:**
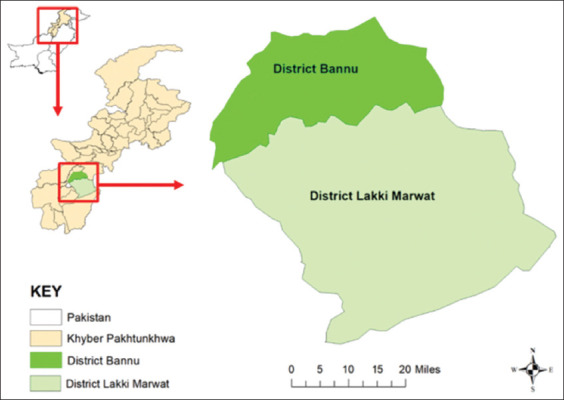
Map depicts the two districts where the study animals were sampled in Khyber Pakhtunkhwa, Pakistan [Source: The map was generated using ArcMap 10.5].

The study was conducted from January to June 2020 in the Bannu division of Khyber Pakhtunkhwa, Pakistan, encompassing districts Bannu and Lakki Marwat (Figure-1).

### Sample collection

The study examined a total of 197 cattle displaying symptoms of anaplasmosis. Employing a random sampling approach, blood samples (3 mL each) were procured from both regions from cattle of varying ages (<2 years, 2–5 years, and >5 years), genders (male and female), and breeds (crossbred and purebred). Blood was aseptically collected in 3 mLEDTA tube (JINGZ, YBK, China) from the jugular vein of cattle exhibiting clinical signs of anaplasmosis.

### Blood sample processing and microscopic analysis

Immediately after collection, a thin blood smear was made on a glass slide, fixed with methanol, and stained using Giemsa staining. The slides were observed under a compound microscope (Olympus CX23, Japan) to identify the *Anaplasma* species using at least 50 fields of view per slide. *Anaplasma* was identified based on established protocols [17]. *Anaplasma* appears as dense, rounded, and deeply stained intraerythrocytic bodies, approximately 0.3–1.0 μm in diameter. In *Anaplasma marginal*e most stained intraerythrocytic bodies are located on or near the margin of the erythrocyte.

### Statistical analysis

Microsoft Excel 2016 (Microsoft Corporation, Washington, USA) was used for data entry, graphical representation, and prevalence computation. The assessment of risk factors utilized the Chi-square test (univariate analysis) and odds ratios (OR) with a 95% confidence interval (CI). These analyses were performed using GraphPad Prism 9 (GraphPad Software Inc., California, USA).

## Results

### Morphological characteristics of recognized *Anaplasma* species

**Figure-2:**
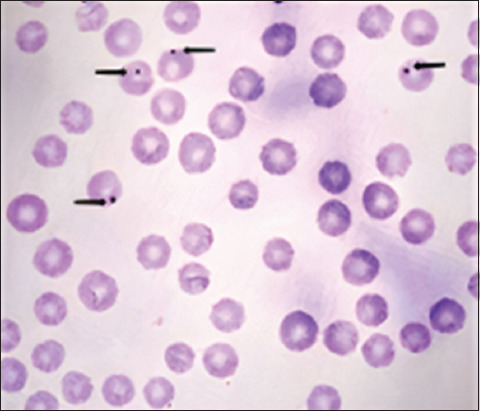
Morphological characteristics of *Anaplasma* inclusions within erythrocytes.

Observation of the Giemsa-stained blood films revealed distinct attributes of *Anaplasma* organisms, such as characteristic features of specific *Anaplasma* strains, the stage of their evolution, and the type of cells they infect. Notably, these features manifested as circular, purplish inclusions ranging from 0.3 to 1 μm in diameter and were consistently present within the erythrocytes from all blood smears. Remarkably dense and uniform, these inclusions assumed a polar or subpolar positioning along the periphery of the erythrocytes (Figure-2).

### The overall prevalence and spatiotemporal variation

This study performed a comprehensive examination of 197 symptomatic cattle, comprising 61 purebred and 136 crossbred specimens, all of which were suspected of anaplasmosis. Of these samples, 39 cattle tested positive for anaplasmosis, resulting in an overall prevalence rate of 19.79%. Based on the geographical distribution, the Bannu district (22.1%) showed higher anaplasmosis occurrence than the Lakki Marwat district (17.64%), although this difference was not statistically significant (χ^2^ = 0.61, p > 0.05). The specific localities within the studied areas did not emerge as significant risk factors (Table-1). Prevalence rates displayed noticeable temporal variation, with the highest recorded in June (45.71%), followed by May (38.7%), April (18.51%), and March (10%). Whereas, the lowest prevalence rates were observed in January (4.34%) and February (3.57%). While the prevalence did not differ significantly between February (reference category), January (χ^2^ = 0.02, p > 0.05), March (χ^2^ = 0.94, p > 0.05), and April (χ^2^ = 3.15, p > 0.05), the prevalence was significantly higher in May (χ^2^ = 10.57, p < 0.05) and June (χ^2^ = 14.02, p < 0.05). In February, the risk of *Anaplasma* infection was 17.05 (95% CI: 2.04–142.4) and 22.73 (95% CI: 2.77–186.3) times lower compared to May and June, respectively (Table-1).

**Table-1:** Spatiotemporal variation of anaplasmosis in cattle analyzed in this study.

Abiotic factors	Variables	Total	Infected	Rates (% ± CI^1^)	Chi-square^2^	OR^3^	p-value
Area	Bannu	95	21	22.1 ± 0.08	0.61	1.32	0.43
	Lakki Marwat	102	18	17.6 ± 0.07			
Month	January	46	2	4.34 ± 0.05	0.02	1.22	0.86
	February	28	1	3.57 ± 0.06	Reference category		
	March	30	3	10.0 ± 0.10	0.94	3.00	0.33
	April	27	5	18.5 ± 0.14	3.15	6.13	0.07
	May	31	12	38.7 ± 0.17	10.57	17.05	0.001*
	June	35	16	45.7 ± 0.16	14.02	22.73	0.000*
Total		197	39	19.7 ± 0.05			

1 CI=Confidence interval,

2 χ^²^=Chi-square,

3 OR=Odd ratios,

*: Significant p-value

### Biotic risk factors analysis

The investigation examined various factors, including cattle breed, locality, gender, month, and age, to identify potential risk factors for anaplasmosis. Univariate analysis highlighted three variables as significant risk factors: Cattle breed and age, and month (Table-2). Prevalence varied between female cattle (22.36%) and male cattle (11.11%), without any significant statistical difference (χ^2^ = 2.77, p > 0.05) (Table-2). Similarly, the prevalence of anaplasmosis was higher in young cattle aged <2 years (26.78%) and those aged 2–5 years (25%), compared to adults aged >5 years (12.35%). While there was no substantial prevalence difference between the age group >5 years (reference category) and 2–5 years (χ^2^ = 3.71, p > 0.05), the prevalence was significantly higher for the age group <2 years (χ^2^ = 4.86, p < 0.05) (Table-2). Notably, a higher prevalence of 23.52% was observed in crossbred cattle compared to purebred cattle (11.47%). A statistically significant association was observed between anaplasmosis and cattle breed (χ^2^ = 3.85, p < 0.05) (Table-2).

**Table-2:** Biotic risk factors associated with anaplasmosis in cattle of District Bannu and Lakki Marwat.

Biotic factors	Variables	Total	Infected	Rates (% ± CI^1^)	Chi-square^2^	OR^3^	p-value
Gender	Female	152	34	22.3 ± 0.06	2.77	2.30	0.09
	Male	45	5	11.1 ± 0.09			
Breed	Crossbreds	136	32	23.5 ± 0.07	3.85	2.37	0.04*
	Purebred	61	7	11.4 ± 0.08			
Age	<2 years	56	15	26.7 ± 0.11	4.86	2.59	0.02*
	2–5 years	52	13	25.0 ± 0.11	3.71	2.36	0.06
	>5 years	89	11	12.3 ± 0.06	Reference category		
Total		197	39	19.7 ± 0.05			

1 CI=Confidence interval,

2 χ^²^=Chi-square,

3 OR=Odd ratios,

* Significant p-value

## Discussion

In this study, a thorough investigation involving 197 cattle revealed an overall anaplasmosis prevalence of 19.79%. Consistently, Kispotta *et al*. [18] reported a prevalence of 18.5% in Dinajpur, Bangladesh. This highlights the consistency of anaplasmosis impact across different regions. Conversely, Samad *et al*. [19] reported anaplasmosis prevalence of 5.95%, which was lower than that reported here. Contrastingly, higher prevalence rates have been documented by other researchers. For instance, Talukdar and Karim [20] and Chowdhury *et al*. [21] from Bangladesh reported anaplasmosis prevalences of 33% and 70%, respectively. The disparities in the reported prevalence can be attributed to several factors, including distinct cattle breeds, geographical locations, environmental disparities, differences in access to veterinary facilities, and variations in sampling periods. These factors collectively contribute to the diversity in observed prevalence rates across various studies. Further research and comparative analyses are crucial for comprehensively understanding the interplay of these factors in influencing anaplasmosis prevalence.

Regarding breed-specific prevalence, although our study revealed a slightly higher prevalence of anaplasmosis among crossbred (23.52%) than purebred cattle (11.47%), the difference was not statistically significant. This observation is consistent with previous research. For instance, Samad *et al*. [19] reported that the prevalence of anaplasmosis was higher in crossbred cattle than in purebred cattle. Chakraborti [22] also documented a higher prevalence among crossbred cattle (32.38%) than among purebred cattle (10.64%).

Echoing our results, Chowdhury *et al*. [21] also suggested that not all cattle breeds exhibit equal susceptibility to anaplasmosis due to inherent resistance against the causative agent. Contrastingly, Kabir *et al*. [23] suggested that the vulnerability to anaplasmosis does not differ significantly among various cattle breeds due to their natural immunity to ticks and adaptability to challenging environments. However, indigenous cattle breeds often encounter prolonged anaplasmosis exposure, their evolutionary adaptation might contribute to their lower prevalence rates [13].

Our findings are consistent with Siddiki *et al*. [24], who highlighted that local cattle breeds display relatively higher resistance than their crossbred counterparts. Additional studies postulate that crossbred cattle tend to receive more meticulous care, which can mitigate their exposure to vectors and subsequent infection [13]. This complex interplay among breed susceptibility, environmental factors, and husbandry practices might underscore the multifaceted nature of anaplasmosis prevalence and its association with different cattle breeds.

In our investigation, the highest prevalence of anaplasmosis was in June (45.71%), followed by May (38.7%), April (18.51%), March (10%), and January (4.34%), while the lowest prevalence was noted in February (3.57%). This is consistent with Taylor *et al*. [25], who reported higher anaplasmosis prevalence during May, June, July, August, September, and October. The seasonal pattern can be attributed to the hot and humid weather conditions, which favor the proliferation and reproduction of insects and ticks, facilitating the transmission of pathogens.

Our results are validated in the work of Atif *et al*. [26] and Khan *et al*. [13] conducted in distinct regions of Pakistan. These studies corroborate the impact of seasonal dynamics on anaplasmosis prevalence, emphasizing the role of environmental factors in influencing the epidemiological patterns of the disease. Such consistency across different investigations underscores the importance of understanding the interplay between weather conditions and disease transmission dynamics for effective disease management and prevention strategies.

This study reported that the incidence of anaplasmosis in females (22.36%) was 2.3 times higher than that in males (11.11%). This aligns with Chakraborti [22], who documented a higher prevalence of anaplasmosis in female cattle (29.71%) compared to their male counterparts (12.5%). Similarly, other studies, including Vetrivel *et al*. [27], Khan *et al*. [13], and Atif *et al*. [26], also reported similar results.

Alim *et al*. [28] suggested a potential link between the host’s gender and the susceptibility and severity of anaplasmosis. They proposed that female cattle are more vulnerable possibly due to pregnancy-related stress and hormonal imbalances. This aligns with Kamani *et al*. [29] and Thrusfield [30], who introduced the concept of pregnancy-induced immunosuppression and the involvement of hormonal dynamics. These factors collectively contribute to the observed gender-based variations in prevalence of anaplasmosis.

In this study, the highest infection rate (26.78%) was documented among cattle aged <2 years followed by those aged 2–5 years (25%). Conversely, the lowest incidence (12.35%) was observed in cattle over 5 years of age. The prevalence among cattle older than 5 years was 2.59 times lower than that observed in cattle below 2 years of age. These findings parallel the outcomes reported by Khan *et al*. [13], Chakraborti [22], and Chowdhury *et al*. [21], who all noted a higher prevalence of anaplasmosis among young cattle than their adult counterparts.

Our results are also consistent with Kispotta *et al*. [18], who reported elevated anaplasmosis occurrences in cattle aged <2 years (20%) than those between 2–4 years (10.86%). The increased prevalence can be attributed to the relatively weaker immune systems in young cattle. Contrastingly, cattle aged over 5 year’s exhibit stronger immunity, which naturally enhances their resistance to several diseases. This association between age and immunity highlights the importance of age-related immunity in influencing disease susceptibility and prevalence patterns.

## Conclusion

This study offers valuable insights into the prevalence of anaplasmosis among cattle in the Bannu and Lakki Marwat districts of Khyber Pakhtunkhwa, Pakistan. We reported significant variations in prevalence of anaplasmosis across factors, including breed, month, gender, and age. However, we should emphasize that our informative study has certain limitations, such as the absence of molecular confirmation of microscopic findings. Despite these limitations, a comprehensive understanding of the multifaceted factors influencing the prevalence of anaplasmosis is crucial for developing effective strategies to manage anaplasmosis. This can safeguard cattle health and minimize economic losses within the livestock industry. Future research with molecular confirmation can further enhance our understanding of this important issue.

## Authors’ Contributions

KU, TU, and NR: Designed this study. FB and MK: Collected samples and epidemiological data and performed the study. MBS and PRD: Performed the statistical analysis. FB, MK and PRD: Drafted the manuscript. MK and MBS: Edited and finalized the manuscript. All authors have read, reviewed, and approved the final manuscript.
